# Revisiting and updating molecular epidemiology of α-thalassemia mutations in Thailand using MLPA and new multiplex gap-PCR for nine α-thalassemia deletion

**DOI:** 10.1038/s41598-023-36840-8

**Published:** 2023-06-17

**Authors:** Wittaya Jomoui, Sitthichai Panyasai, Pornpun Sripornsawan, Wanicha Tepakhan

**Affiliations:** 1 Department of Pathology, Faculty of Medicine, Maha Chakri Sirindhorn Medical Center, Srinakharinwirot University, Nakhon Nayok, 26120 Thailand; 2 Department of Medical Technology, School of Allied Health Sciences, University of Phayao, Phayao, 56000 Thailand; 3 Department of Pediatrics, Faculty of Medicine, Prince of Songkla University, Songkhla, 90110 Thailand; 4 Department of Pathology, Faculty of Medicine, Prince of Songkla University, Songkhla, 90110 Thailand

**Keywords:** Clinical genetics, Medical genetics, Mutation, Population genetics

## Abstract

α-thalassemia is an inherited blood disorder that is most frequently found in Southeast Asian populations. In Thailand, molecular characterization can diagnose most patients with α-thalassemia; however, several atypical patients are also observed in routine analyses. Here, we characterized α-thalassemia mutations among 137 Hemoglobin H (Hb H) disease patients and three fetuses of Hb Bart’s hydrops, a fatal clinical phenotype of α-thalassemia. Specifically, we performed multiplex ligation-dependent probe amplification (MLPA) followed by direct DNA sequencing. We noticed common genotypes in 129 patients and eight patients had rare Hb H disease caused by compound heterozygous α^0^-thalassemia (--^CR^ or --^SA^ deletion) with α^+^-thalassemia (-α^3.7^/-α^4.2^/α^Constant Spring^α). Furthermore, two affected fetuses had the --^SA^/--^SEA^ and one had the --^CR^/--^SEA^ genotypes. Next, we developed and validated a new multiplex gap-PCR and applied this method to 844 subjects with microcytic red blood cells (RBCs) from various parts of Thailand. The frequency of heterozygous α^0^-thalassemia was dominated by --^SEA^ 363/844 (43%), followed by --^THAI^ 3/844 (0.4%), --^SA^ 2/844 (0.2%), and --^CR^ 2/844 (0.2%) mutations. These findings suggest that aforementioned four mutations should be routinely applied to increase the effectiveness of diagnosis and genetic counseling in this region.

## Introduction

α-Thalassemia is caused by mutations in the α-globin gene (*HBA*) on chromosome 16, resulting in reduced or absent α-globin chain production called α^+^-thalassemia (-α or α^T^α) and α^0^-thalassemia (--), respectively. α^0^-thalassemia is usually caused by large deletions covering both *HBA2* and *HBA1* genes; however, α^+^-thalassemia can be caused by either an additional mutation that affects only a single *HBA* gene or a point mutation that reduces α-globin chain production. Heterozygous α-thalassemia has no clinical symptoms and only presents with hypochromic microcytic red blood cells (RBCs). However, compound heterozygous α^0^-thalassemia with α^+^-thalassemia leads to hemoglobin H (Hb H) disease, which produces moderate-to-severe anemia depending on the type of α^+^-thalassemia mutation. For example, deletional Hb H disease (--/-α) causes less severe anemia than non-deletional version (--/α^T^α)^1,2^. Moreover, homozygous α^0^-thalassemia (--/--) results in a severe thalassemia disease called hemoglobin Bart (Hb Bart)’s hydrops fetalis. Hb Bart’s hydrops disease produces severe clinical manifestations from the fetal stage, such as severe anemia, generalized edema, and pleural and pericardial effusions, often resulting in fetal or neonatal mortality worldwide^3^. In Thailand, α-thalassemia carriers have been reported to be as high as 30–40%^4^. The common α^0^-thalassemia mutations are --^SEA^ (NC_000016.10:g.165397_184700) and --^THAI^ deletion (NC_000016.10:g.149863_183312), and α^+^-thalassemia mutations were -α^3.7^ (NG_000006.1:g.34164_37967del3804), -α^4.2^ (NC_000016.10:g.169818_174075del), Hb Constant Spring (CS, HBA2:c.427 T > C), and Hb Paksé (PS, HBA2:c.429A > T)^5^. In Thailand, a total of 423 Hb Bart’s hydrops fetalis stillbirths and 3,172 newborns with Hb H disease were estimated in 2020 ^6^. Thus, α-thalassemia remains a crucial public health concern in Thailand.

Molecular diagnosis is routinely performed for the prevention and control of severe α-thalassemia. However, a majority of laboratories focus on identifying six common mutations, including four deletional mutations (--^SEA^, --^THAI^, -α^3.7^, -α^4.2^) and two non-deletional mutations (Hb CS and Hb PS)^7,8^. Although most patients are assigned α-thalassemia diseases based on molecular diagnosis, a few of them are categorized as suffering from Hb Bart’s hydrops fetalis or Hb H disease, even though genotypes remain unknown. Therefore, to increase the effectiveness of the prevention and control program for severe α-thalassemia in this region, in addition to common α-thalassemia mutations, rare or unknown mutations should also be identified. Here, using the multiplex ligation-dependent probe amplification (MLPA) technique, we report a spectrum of common Hb H diseases and identification of previously unknown Hb H genotypes and Hb Bart’s hydrops fetalis cases from southern Thailand. To update the α-thalassemia genotype in Thailand, multiplex gap-polymerase chain reaction (gap-PCR) of common and rare mutations observed in this study was developed and validated for application in the screening of α-thalassemia among the Thai population with microcytic RBCs.

## Material and methods

All methods were carried out in accordance with relevant guidelines and regulations. Ethical approval of the study protocol was obtained from the Human Research Ethics Unit (HREU), Faculty of Medicine, Prince of Songkla University (REC 64-054-5-2). The Human Research Ethics Unit, Faculty of Medicine, Prince of Songkla University, allow us to waive the requirement to obtain any informed consent because we use unidentifiable leftover specimens.

### Specimens

Leftover DNA samples after routine molecular diagnosis from three unrelated families with unknown Hb Bart’s hydrops fetalis were collected. A total of 137 DNA samples, either common Hb H disease or uncharacteristic Hb H disease, were obtained from the Thalassemia Unit, Department of Pathology, Faculty of Medicine, Prince of Songkla University, southern Thailand.

For the study of the genotype and spectrum of α-thalassemia in the Thai population with microcytic RBCs, a total of 844 leftover DNA samples with mean corpuscular volume (MCV) levels < 80 fL, Dichlorophenol Indophenol Precipitation (DCIP) negative and hemoglobin (Hb) pattern showed only Hb A and Hb A2 < 3.5% were selected from three laboratory centers in Thailand. These included 232 subjects from the Department of Pathology, Faculty of Medicine, Maha Chakri Sirindhorn Medical Center, Srinakharinwirot University (central and east); 422 subjects from the School of Allied Health Science, University of Phayao (north); and 190 subjects from the Department of Pathology, Faculty of Medicine, Prince of Songkla University (south).

### Hematological analysis

Hematological parameters were recorded from each center based on the national guidelines in Thailand^9^. Hb profiles were determined using capillary electrophoresis (CE) (Capillarys 2; Sebia, Lisses, France).

### DNA analysis for α-thalassemia

Identification of α-thalassemia mutations, such as --^SEA^, --^THAI^, --^MED^ (NG_000006.1:g.24664_41064del16401), --^FIL^ (NG_000006.1:g.11684_43534del31851), -(α)^20.5^ (NG_000006.1:g.15164_37864del22701), -α^3.7^, -α^4.2^, Hb CS, and Hb PS, was performed using the PCR technique as previously described^7,8,10^. Uncharacterized common α-thalassemia mutations in Hb H disease and Hb Bart’s hydrops fetalis samples were further scanned for unknown mutations using MLPA and direct DNA sequencing.

### MLPA analysis and direct DNA sequencing

Dosage quantitative analysis of the α-globin gene cluster on chromosome 16p13.3 was carried out in an unidentified Hb H disease and Hb Bart’s hydrops fetalis subjects using an MLPA commercial kit (SALSA MLPA probemix P140-C1 HBA, MRC-Holland, Amsterdam, The Netherlands). MLPA analysis was performed on a SimpliAmp™ thermal cycler (Thermo Fisher Scientific, Waltham, MA, USA) according to the manufacturer’s instructions. Fragment analysis was performed using an ABI PRISM™ 3500 analyzer (Applied Biosystems, Foster City, CA, USA). Data analysis was performed using the Coffalyser.Net™ software (MRC Holland). Gap-PCR to detect rare α-thalassemia genotypes (--^SA^ deletion (NG_000006.1:g.19464_43064del23601) and --^CR^ deletion (NC_000016.10:g.144,215_188,841)) was conducted after receiving the MLPA results, as previously identified^11,12^. Breakpoint confirmation of targeted PCR fragments was performed by direct DNA sequencing using an ABI PRISM™ 3130 XL analyzer (Applied Biosystems).

### Development of a multiplex gap-PCR for diagnosis of the common and rare α-thalassemia deletion mutations

A multiplex gap-PCR for genotyping nine α-thalassemia deletion mutations, including --^SEA^, --^THAI^, --^FIL^, --^MED^, -(α)^20.5^, --^SA^, --^CR^, -α^3.7^, and -α^4.2^ in a single tube was developed. Eleven PCR primer pairs from previous studies^7,10,13,14^ were used to amplify ten specific PCR fragments and one internal control fragment (Table 1). The PCR mixture containing 50–200 ng genomic DNA, 1.3 μM each of LIS1-F, LIS1-R, FIL-F, and FIL-R primers, 1 μM each of SA-F and SA-R primers, 0.8 μM of 3.7/20.5-R primers, 0.7 μM each of α2/3.7-F, 4.2-F, and 4.2-R primers, 0.4 μM each of SEA-F and SEA-R primers, 0.3 μM each of MED-F, MED-R, THAI-F, THAI-R, α2-R, 20.5-F, CR-F, and CR-R primers, 200 μM of each dNTP, 1.5 mM MgCl_2_, 10 mM DTT, 1 M Betaine, 1 Unit GoTaq Flexi DNA polymerase (Promega Corporation, Madison, WI, USA) in 1 × Green GoTaq buffer (50 mM Tris–HCl (pH 9.0) buffer, 50 mM NaCl, 5 mM MgCl_2_), sterile distilled water in a final volume of 30 μL. PCR amplification was performed using a SimpliAmp™ thermal cycler (Thermo Fisher Scientific). After initial heating at 97 °C for 5 min, the reaction was followed by 26 cycles at 97 °C for 1 min, 61 °C for 3 min, and 72 °C for 2 min, and a final extension at 72 °C for 5 min. The amplified fragments were separated using 1.5% agarose gel electrophoresis for 45 min in 0.5 × TAE buffer. After ethidium bromide staining, the PCR products were visualized under UV light.

**Table 1:** Primers for single-tube multiplex gap-PCR analysis of nine α-thalassemia deletion mutations.

Primer name	Primer sequence (5′–3′)	Amplicon size	References
LIS1-F	GTCGTCACTGGCAGCGTAGATC	LIS1 3’UTR fragment (2503 bp)	^7^
LIS1-R	GATTCCAGGTTGTAGACGGACTG		^7^
α2/3.7-F	CCCCTCGCCAAGTCCACCC	-α^3.7^ Junction fragment (2,022/2,029 bp)	^7^
3.7/20.5-R	AAAGCACTCTAGGGTCCAGCG		^7^
α2/3.7-F	As above	α2 gene (1800 bp)	^7^
α2-R	AGACCAGGAAGGGCCGGTG		^7^
4.2-F	GGTTTACCCATGTGGTGCCTC	-α^4.2^ Junction fragment (1628 bp)	^7^
4.2-R	CCCGTTGGATCTTCTCATTTCCC		^7^
SEA-F	CGATCTGGGCTCTGTGTTCTC	--^SEA^ Junction fragment (1349 bp)	^7^
SEA-R	AGCCCACGTTGTGTTCATGGC		^7^
THAI-F	GGCACTGAGAGCCCTTCACG	--^THAI^ Junction fragment (1024 bp)	^10^
THAI-R	CAAGTGGGCTGAGCCCTTGAG		^10^
20.5-F	GCCCAACATCCGGAGTACATG	-(α)^20.5^ Junction fragment (1007 bp)	^7^
3.7/20.5-R	As above		^7^
MED-F	TACCCTTTGCAAGCACACGTAC	--^MED^ Junction fragment (807 bp)	^7^
MED-R	TCAATCTCCGACAGCTCCGAC		^7^
FIL-F	TGCAAATATGTTTCTCTCATTCTGTG	--^FIL^ Junction fragment (1166 bp)	^7^
FILR	ATAACCTTTATCTGCCACATGTAGC		^7^
CR-F	TAGCCTGGAGGACAGAGTAAG	--^CR^ Junction fragment (335 bp)	^14^
CR-R	CGGGTCATGTTGAGTAGGAATAA		^14^
SA-F	CTCATCCAGACTCTCCAGCT	--^SA^ Junction fragment (1129 bp)	^13^
SA-R	ACTCGAGCTACCCCAAGGAT		^13^

## Results

### Identification of unknown Hb H disease and unknown Hb Bart’s hydrops fetalis

**Figure 1:**
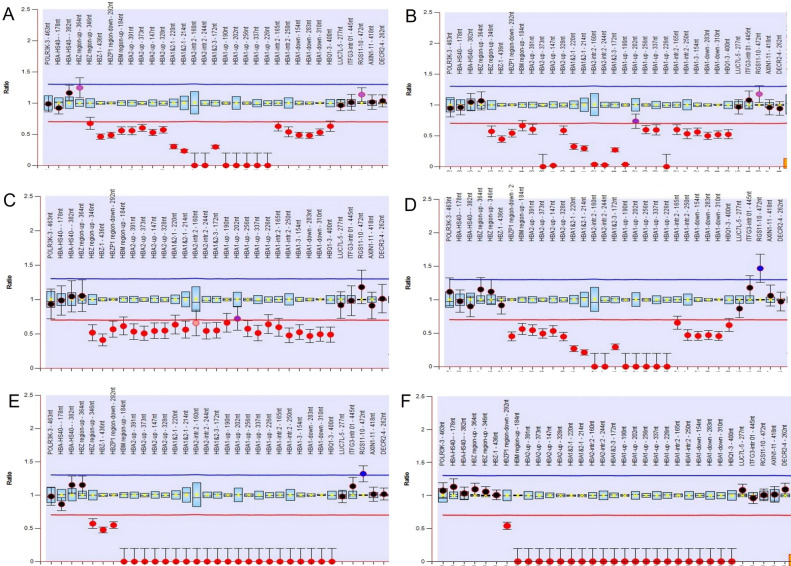
MLPA analysis among unknown Hb H disease and unknown Hb Bart’s hydrops fetalis patients from southern Thailand. After mutation confirmation, the MLPA pattern of each patient was completed; the genotypes included compound heterozygous --^CR^/-α^3.7^ (**A**), compound heterozygous --^CR^/-α^4.2^ (**B**), compound heterozygous --^CR^ with Hb Constant Spring (**C**), compound heterozygous --^SA^/-α^3.7^ (**D**), compound heterozygous --^CR^/--^SEA^ (**E**), and compound heterozygous --^SA^/--^SEA^ (**F**).

The molecular diagnosis of α-thalassemia mutations among 137 Hb H disease patients revealed five common genotypes in 129 (94.2%) and unknown genotypes in 8 (5.8%) subjects. The common Hb H genotypes comprised 86 (62.8%) patients with --^SEA^/-α^3.7^, 5 (3.6%) patients with --^SEA^/-α^4.2^, 1 (0.7%) patient with --^THAI^/-α^3.7^, 35 (25.5%) patients with --^SEA^/α^CS^α, and 2 (1.5%) patients with --^SEA^/α^PS^α (Table 2). The remaining eight subjects were classified as having unknown Hb H genotypes based on their Hb H or Hb Bart's levels; however, routine molecular diagnosis revealed homozygous α^+^-thalassemia. Among these, seven cases were unknown deletional Hb H disease, and one was unknown non-deletional Hb H disease. MLPA was used to scan unknown deletion sizes in the α-globin gene cluster. The results showed that six patients had the same large functional α^0^-thalassemia covering probes 5–28. Two of them were combined with α^+^-thalassemia (-α^3.7^), three with α^+^-thalassemia (-α^4.2^), and one with Hb CS (Fig. 1A–C). The MLPA results of the remaining two patients showed the same large functional α^0^-thalassemia covering probes 7–28. Both were combined with α^+^-thalassemia (-α^3.7^) (Fig. 1D), but one patient also co-inherited Hb C heterozygosity. Interestingly, the MLPA pattern of these two unknown α^0^-thalassemia cases was reported and the deletion breakpoint was confirmed as a Chiang Rai deletion (--^CR^, deletion from probes 5–28) and a South Africa deletion (--^SA^, deletion from probes 7–28) in previous studies^11–13^. This suggests that these two cases of α^0^-thalassemia might involve the same mutations observed in this study. Thus, gap-PCR for --^CR^ and --^SA^ deletions was performed, followed by direct DNA sequencing. The DNA sequencing results showed the same deletion breakpoints for the --^CR^ deletion and --^SA^ deletion, as previously reported. Thus, the genotypes of eight unknown Hb H disease patients in this study consisted of two cases with --^CR^/-α^3.7^, three cases with --^CR^/-α^4.2^, one case with --^CR^/α^CS^α, and two cases with --^SA^/-α^3.7^ genotypes. Table 2 shows the hematological parameters and genotype frequencies of the 137 patients with Hb H disease.

**Table 2:** Genotype frequency and hematological parameters of common and rare HbH diseases in southern Thailand.

Genotype	Frequency (%)	n	Age (Years)	Hb (g/dL)	Hct (%)	MCV (fL)	MCH (pg)	RDW (%)	Hb A2 (%)	Hb H (%)	Hb Bart's (%)	Hb F (%)	Hb CS (%)	Hb C (%)
Common	94.2	129												
--^SEA^/-α^3.7^	62.8	7	0.5 m ± 0.7	10.2 ± 2.4	33.0 ± 6.6	63.1 ± 11.0	19.4 ± 3.9	24.4 ± 2.1	0.9 ± 0.1	1.6 ± 1.4	13.0 ± 10.0	34.3 ± 28.8		
		79	25.1 ± 19.7	8.6 ± 1.2	28.4 ± 4.2	57.5 ± 6.0	17.3 ± 1.5	24.8 ± 2.7	1.6 ± 0.5	6.1 ± 3.8	2.1 ± 1.6	4.2 ± 3.5		
--^SEA^/-α^4.2^	3.6	5	27 ± 14.7	8.9 ± 1.9	31.2 ± 4.1	67.4 ± 10.6	18.8 ± 1.7	27.0 ± 5.9	1.3 ± 0.5	10.3 ± 5.2	1.8 ± 0.7			
--^THAI^/-α^3.7^	0.7	1	9	6.9	21.9	54	17	20.2	1.3	4.8	0.8			
--^SEA^/α^CS^α	25.5	35	16.1 ± 18.6	7.4 ± 1.8	26.1 ± 6.8	66.8 ± 11.4	19.3 ± 3.6	24.9 ± 3.9	1.3 ± 0.6	11.7 ± 5.4	5.7 ± 2.5	1.5 ± 0.8	2.2 ± 0.8	
--^SEA^/α^PS^α	1.45	2	NA, 8	6.4, 10.2	33.0, 30.3	63.0, 57.0	12.2, 19.2	17.4, 29.6	1.2, 1.9	15.9, 7.5				
Unknown	5.8	8												
--^CR^/-α^3.7^	1.45	2	6, 21	7.8, 8.2	26.9,27.2	57.0, 54.0	16.6, 15.9	28.5, 22.0	1.6, 1.6	5.9, 4.2	0, 6.8			
--^CR^/-α^4.2^	2.2	2	0 m, 3 m	10.9, 8.2	36.3, 28.3	75.3, 63.7	22.6, 18.5	23.2, 22.5	0	0.3, 0.8	25.0, 17.4	48.3, 28.6		
		1	40	9	28.8	57	17.8	26.7	1.6	4.1	8.9			
--^SA^/-α^3.7^	1.45	1	24	7.5	25.8	67	19.5	15.4	1.3	8.9				
--^SA^/-α^3.7^, β^C^/β		1	2	8.7	28.3	46	14.2	26.5	3.5		0.8			19.4
--^CR^/α^CS^α	0.7	1	26	7.8	29.6	78	20.6	27.6	0.9	2.7	4	2.4	0.9

This study also included three unrelated families with unknown Hb Bart’s hydrops fetalis. All three parents presented with very low MCV levels (< 70 fL), but DNA analysis found only α^0^-thalassemia (--^SEA^) carriers in one individual (Table 3). Thus, they were diagnosed as non-couples at risk of Hb Bart’s hydrops fetalis. However, the fetus presented with severe anemia and hydrops during gestation. Fetal cord blood was collected, and Hb analysis was performed, followed by re-identification of common α^0^-thalassemia in DNA samples from cord blood and their parents. Hb analysis of fetal cord blood samples revealed Hb Bart's levels with embryonic Hb levels of 100%. Common α^0^-thalassemia genotype results were detected in the parents indicating the same results as previous identification attempts, but DNA analysis of fetal samples showed the homozygous α^0^-thalassemia (--^SEA^) genotype. This suggested that the affected fetus was Hb Bart’s hydrops fetalis from a compound heterozygous α^0^-thalassemia mutation (--^SEA^/--^Unknown^). Fetal DNA samples were scanned for unknown mutations using the MLPA method. The results showed that a fetus from family I presented a large deletion from probes 5–28, which was suspected to be the --^CR^ deletion (Fig. 1E). Two fetuses from families II and III presented a large deletion from probes 7–28, which were suspected to be the --^SA^ deletions (Fig. 1F). Gap-PCR was performed, and the deletion breakpoint was confirmed by direct DNA sequencing. DNA sequencing results demonstrated that the fetus from family I was compound heterozygous for α^0^-thalassemia (--^SEA^/--^CR^). Family II and III fetuses were compound heterozygous α^0^-thalassemia (--^SEA^/--^SA^).

**Table 3:** Hematological parameters and α-thalassemia genotypes in three families with unknown Hb Bart’s hydrops fetalis in southern Thailand. F; Family, M; Mother, Fa; Father, P; Proband, NA; Not available.

Parameter	FI-M	FI-Fa	FI-P	FII-M	FII-Fa	FII-P	FIII-M	FIII-Fa	FIII-P
Hb (g/dL)	11.4	12.6	NA	11.0	14.6	5.4	10.7	13.5	5.3
Hct (%)	36.5	41.3	NA	34.2	43.7	21.6	33.7	43.3	25.9
MCV (fL)	67.0	60.3	NA	68.0	63.0	99.1	64.0	64.0	118.8
MCH (pg)	20.9	NA	NA	21.7	20.9	24.8	20.4	20.1	24.3
RDW (%)	17.9	NA	NA	19.3	19.9	42.8	16.3	17.1	30.3
Hb type	A2A	A2A	NA	A2A	A2A	Bart’s and embryonic Hb	A2A	A2A	Bart’s and embryonic Hb
Hb A2 (%)	2.5	5.2	NA	2.0	2.0	0	2.2	2.4	0
Genotype	--^CR^/αα	--^SEA^/αα	--^CR^/--^SEA^	--^SEA^/αα	--^SA^/αα	--^SA^/--^SEA^	--^SA^/αα	--^SEA^/αα	--^SA^/--^SEA^

### Accuracy of the developed multiplex gap-PCR assay

**Figure 2:**
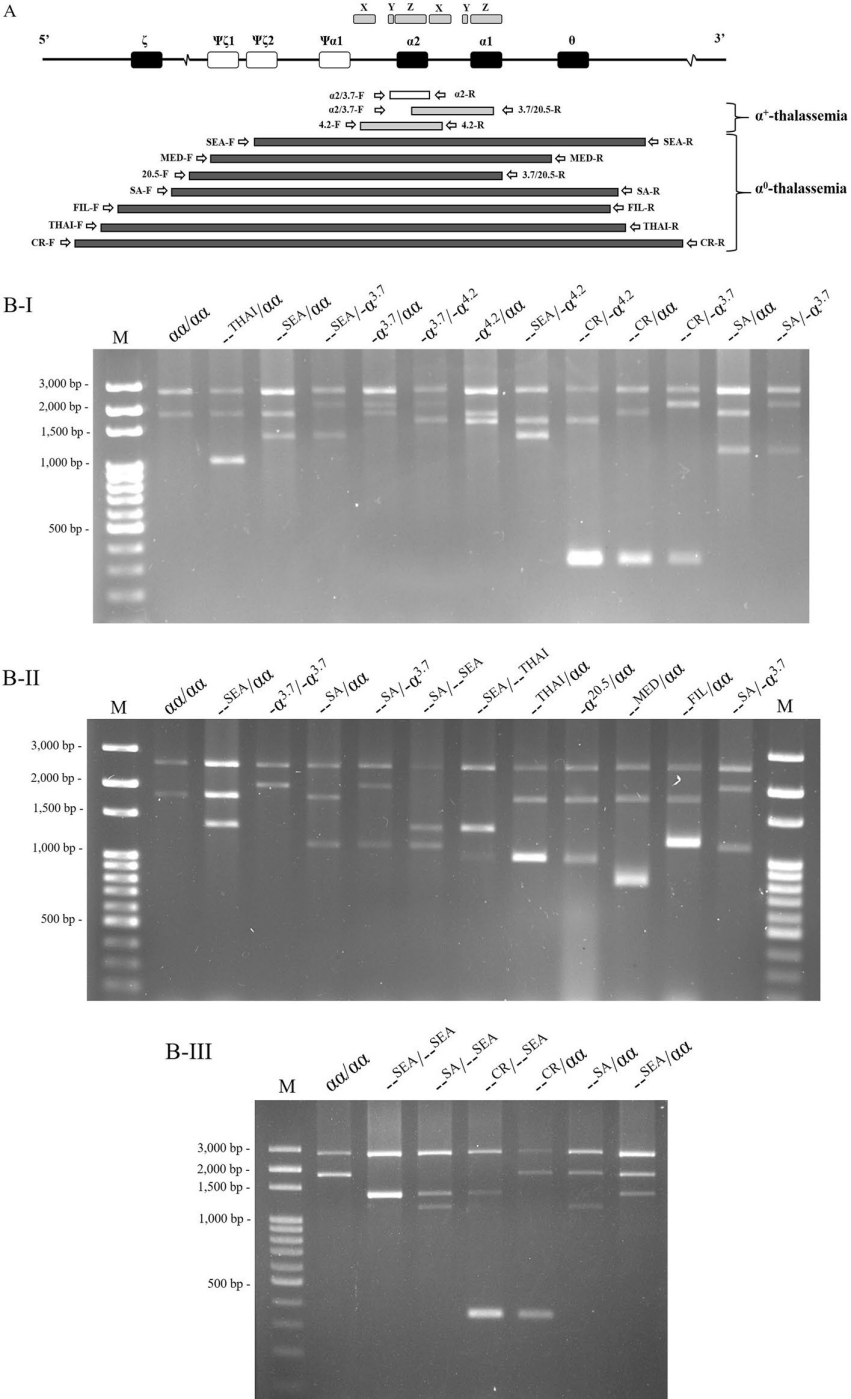
Schematic diagram of primer orientation for a newly developed multiplex gap-PCR of nine deletional α-thalassemia mutations (**A**) and agarose gel electrophoresis results of 21 α-thalassemia genotypes (**BI–III**). Original gels are presented in Supplementary Fig. 1.

In many cases of uncommon α^0^-thalassemia mutations, --^CR^ and --^SA^ deletions, occurred with Hb H disease and Hb Bart’s hydrops fetalis. Consequently, we developed a multiplex gap-PCR including these two mutations. We also investigated the accuracy of the newly developed multiplex gap-PCR with a total of 35 DNA samples with 21 different α-thalassemia genotypes such as αα/αα, --^SEA^/αα, --^THAI^/αα, --^MED^/αα, --^FIL^/αα, -(α)^20.5^/αα, --^CR^/αα, --^SA^/αα, -α^3.7^/αα, -α^4.2^/αα, -α^3.7^/-α^3.7^, -α^3.7^/-α^4.2^, --^SEA^/-α^3.7^, --^SEA^/-α^4.2^, --^CR^/-α^3.7^, --^CR^/-α^4.2^, --^SA^/-α^3.7^, --^SEA^/--^SEA^, --^SEA^/--^THAI^, --^SEA^/--^CR^, and --^SEA^/--^SA^. Some rare α-thalassemia genotypes that have only one sample were split into triplicated samples before validating the method. The DNA samples were blinded before testing with multiplex gap-PCR by two laboratory staff. The agarose gel electrophoresis results for all 21 genotypes are shown in Fig. 2 (original gels are presented in Supplementary Fig. 1). The results of our developed method were compared with the results of routine multiplex gap-PCR and MLPA with DNA sequencing for --^CR^ and --^SA^ deletions. Our newly developed multiplex gap-PCR method showed 100% concordance with the reference methods. Thus, this newly developed method was applicable for further investigation of α-thalassemia frequency in the Thai population with microcytic RBCs.

### Genotype and frequency of common and rare α-thalassemia in Thailand

According to the Prevention and Control Program for Severe Thalassemia, the national screening criteria for suspected α^0^-thalassemia carriers involves using a cut-off point of MCV < 80 fL, DCIP negative, and Hb pattern presented with only Hb A and Hb A2 < 3.5%^15,16^. Thus, to study the frequency of --^CR^ and --^SA^ deletion in the Thai population, a total of 844 specimens was collected from four parts of the country including northern (n = 422), central and eastern (n = 232), and southern (n = 190) regions, based on those screening criteria. All DNA samples were identified the α-thalassemia genotype using our newly developed multiplex gap-PCR for nine α-thalassemia mutations and allele specific PCR (ASPCR) for Hb CS and Hb PS, as described previously^8^. The results in Table 4 demonstrate that deletional α^0^-thalassemia heterozygote (--/αα) was frequently observed in 370/844 subjects (43.8%). The most frequent mutation was --^SEA^ deletion (363/844, 43.0%), followed by --^THAI^ deletion (3/844, 0.4%), --^SA^ deletion (2/844, 0.2%), and --^CR^ deletion (2/844, 0.2%). The --^SEA^ deletion was frequently found in populations from all four parts of Thailand and dominated the northern population. For rare deletional α^0^-thalassemia, the --^THAI^ deletion was found in populations from the central, eastern, and northern regions, but not in the southern population. Interestingly, --^SA^ and --^CR^ deletions were identified only in the southern population. The deletional α^+^-thalassemia heterozygote (-α/αα) was the second common genotype (178/844, 21.1%). The most frequent mutation was the -α^3.7^ deletion (167/844, 19.8%), followed by the -α^4.2^ deletion (11/844, 1.3%). These two mutations were observed across all the populations in this study. However, these genotypes showed prevalence in the central, eastern, and southern populations. The third most frequent genotype (65/844, 7.7%) was the deletional α^+^-thalassemia homozygote or compound heterozygote genotype (− α/− α). The most common genotype was -α^3.7^/-α^3.7^ (58/844, 6.9%) followed by -α^3.7^/-α^4.2^ (6/844, 0.7%), and -α^4.2^/-α^4.2^ (1/844, 0.1%), respectively. The non-deletional α^+^-thalassemia heterozygote was the latter genotype (40/844, 4.7%). Heterozygous Hb CS was identified in 39/844 (4.6%) of the four population groups, and heterozygous Hb PS was identified in only 1/844 (0.1%) of the central population. The last genotype observed in this study was compound heterozygous α^+^-thalassemia (α^CS^α/-α^3.7^), with a frequency of 12/844 (1.4%). This genotype was predominantly identified in the northern population and rarely identified in the central and eastern populations.

**Table 4:** Frequency of α-thalassemia genotypes among 844 subjects with MCV < 80 fL and hemoglobin profiles showed A2A pattern with Hb A2 < 3.5% from each part of Thailand.

Genotype	Total no.	(%)	Part of Thailand					
			Central and East		North		South	
			n	(%)	n	(%)	n	(%)
--^SEA^/αα	363	(43.0)	60	(25.9)	240	(56.9)	63	(33.2)
--^THAI^/αα	3	(0.4)	1	(0.4)	2	(0.5)	0	(0.0)
--^SA^/αα	2	(0.2)	0	(0.0)	0	(0.0)	2	(1.1)
--^CR^/αα	2	(0.2)	0	(0.0)	0	(0.0)	2	(1.1)
-α^3.7^/αα	167	(19.8)	71	(30.6)	42	(10.0)	54	(28.4)
-α^4.2^/ αα	11	(1.3)	4	(1.7)	5	(1.2)	2	(1.1)
-α^3.7^/-α^3.7^	58	(6.9)	13	(5.6)	37	(8.8)	8	(4.2)
-α^4.2^/-α^4.2^	1	(0.1)	0	(0.0)	1	(0.2)	0	(0.0)
-α^3.7^/-α^4.2^	6	(0.7)	1	(0.4)	4	(1.0)	1	(0.5)
α^CS^α/αα	39	(4.6)	4	(1.7)	30	(7.1)	5	(2.6)
α^PS^α/αα	1	(0.1)	1	(0.4)	0	(0.0)	0	(0.0)
α^CS^α/-α^3.7^	12	(1.4)	1	(0.4)	11	(2.6)	0	(0.0)
α^CS^α/-α^4.2^	0	(0.0)	0	(0.0)	0	(0.0)	0	(0.0)
αα/αα	179	(21.2)	76	(32.8)	50	(11.9)	53	(27.9)
Total	844	100.0	232	100.0	422	100.00	190	100.0

## Discussion

In southern Thailand, the frequency of --^SEA^ mutation in couples at risk of having Hb Bart’s hydrops fetalis is reported to be as high as 98%^17^. However, our study demonstrated that several families were misdiagnosed as not at risk of having Hb Bart’s hydrops fetalis because they were carriers of another rare α^0^-thalassemia mutation that could not be identified in routine molecular diagnosis. We identified Hb H disease patients at a rate as high as 5.8%. MLPA, followed by direct DNA sequencing, revealed that unknown Hb Bart’s hydrops fetalis and unknown Hb H diseases were caused by a common α-thalassemia mutation in combination with α^0^-thalassemia (--^CR^ or --^SA^ deletion). Our study demonstrates that an advanced molecular technique, such as MLPA, for the identification of unknown large deletions may increase the efficiency of molecular diagnosis. However, after screening for deletion size by MLPA, gap-PCR for the deletion breakpoint confirmation could not be amplified. Therefore, a specific method, such as next-generation sequencing, should be applied for identification^18^.

The α^0^-thalassemia Chiang Rai deletion (--^CR^ or --^44.6 kb^) was first reported in Thailand from a patient in Chiang Rai province, northern Thailand^12^. However, this mutation was originally identified in two siblings from southern China^19^. In Thailand, the --^CR^ deletion has been reported in several studies involving northern populations. Notably, co-inheritance of the --^CR^ deletion with other α^0^-thalassemia or α^+^-thalassemia alleles results in Hb Bart’s hydrops fetalis (--^SEA^/--^CR^) and Hb H disease (--^CR^/-α^3.7^), respectively^12,20^. Our study is the first report of southern Thailand patients with the same genotype, both Hb Bart’s hydrops fetalis (--^SEA^/--^CR^), and Hb H disease (--^CR^/-α^3.7^). Moreover, we report for the first time, genotypes such as Hb H disease (--^CR^/-α^4.2^) and Hb H CS disease (--^CR^/α^CS^α). The clinical phenotype of deletional Hb H disease (--^CR^/-α^3.7^ and --^CR^/-α^4.2^) was similar to previously reported^20^ deletional Hb H genotypes and involved moderate anemia without requiring transfusion (Table 2). Non-deletional Hb H disease patients had compound heterozygous α^0^-thalassemia (--^CR^) with Hb CS, and presented with moderate to severe anemia and required regular blood transfusions (1–2 units) every 4–5 months. Furthermore, non-deletional Hb H genotype (--^CR^/α^CS^α) showed a stronger clinical severity (Table 2) than that of deletional Hb H genotypes (--^CR^/-α^3.7^ and --^CR^/-α^4.2^). This observation can be explained by the severe instability of Hb CS molecules, resulting in increased precipitation in the RBC membranes. In agreement, the RBCs of non-deletional Hb H disease patients are reported to be more rigid and fragile than those of deletional Hb H disease patients^21^.α^0^-thalassemia South Africa (--^SA^) was originally reported in the South African population and was caused by a large deletion of approximately 23 kb with 158 base insertions, which removed both *HBA2* and *HBA1* genes^22^. Interestingly, the co-inheritance of α^0^-thalassemia (--^SA^) with α^+^-thalassemia was reported in 13/17 Hb H disease, suggesting that this mutation is prevalent in the Indian population^13^. Recently, the first case of rare Hb H disease, caused by compound heterozygous α^0^-thalassemia (--^SA^) with α^+^-thalassemia (-α^3.7^), was reported in northern Thailand^11^. In this study, we found a rare deletional Hb H disease (-^SA^/-α^3.7^) in two individuals from southern Thailand. The hematological parameters of one patient with the --^SA^/-α^3.7^ genotype differed from those previously reported^11^. The lower Hb levels (7.5 g/dL) in this patient may have been caused by increased oxidative stress, resulting in a hemolytic crisis.

In this study, we also report the complex interaction of deletional Hb H (--^SA^/-α^3.7^) with Hb C heterozygote for the first time. Hb C (HBB:c.19G > A) is a missense mutation caused by a single-base substitution from GAG > AAG at codon 6 in the β-globin (*HBB*) gene. Usually, heterozygous Hb C individuals are asymptomatic with normocytic or slightly microcytic RBCs^23,24^ and hemoglobin analysis determined the Hb C levels in a carrier as 37.2 ± 3.2%^24^. Our CE and direct DNA sequencing of the *HBB* gene diagnosed the deletional Hb H (--^SA^/-α^3.7^) with Hb C heterozygote patient as heterozygous Hb C. However, based on observations such as moderate anemia (Hb 8.7 g/dL), very low MCV levels (46 fL) with increased RDW (26.5%), and lower Hb C levels (19.4%) with Hb Bart’s (0.8%), we suspected an abnormality in the *HBA* gene. Routine gap-PCR revealed homozygous α^+^-thalassemia (-α^3.7^) in this patient. However, the Hb Bart's presented by CE suggested that this abnormality affects at least three *HBA* genes. Thus, to obtain a true genotype, we further carried out MLPA and gap-PCR, followed by direct sequencing. We noticed a combination of Hb C heterozygosity with deletional Hb H (--^SA^/-α^3.7^) disease, which did not increase the clinical severity. The hematological parameters also showed a similar result with moderate anemia as compared with other deletional Hb H diseases with a normal β-globin genotype (Table 2). This might be explained by the structural change of the Hb C molecule, which does not affect stability and protein function.

In this study, rare α^0^-thalassemia mutations (--^CR^ and --^SA^) were responsible for multiple cases of Hb Bart’s hydrops fetalis and Hb H disease. This observation suggests that the identification of only two common α^0^-thalassemia mutations (--^SEA^ and --^THAI^)^17,25,26^ is likely insufficient for a prevention and control program for severe thalassemia in Thailand. Thus, we developed a new multiplex gap-PCR method to detect nine α-thalassemia mutations which yielded 100% concordance. However, the α^0^-thalassemia --^THAI^ and -(α)^20.5^ deletions are quite difficult to interpret because of similar amplicon sizes of 1024 bp and 1007 bp, respectively (Fig. 2). Although the α^0^-thalassemia --^THAI^ deletion is more frequently observed in the Thai population than the -(α)^20.5^ deletion (Table 4), a monoplex gap-PCR should be carried out to confirm these mutations.

A rare α^0^-thalassemia mutation (--^CR^ and --^SA^) was identified in the Thai population a few years ago^11,12,20^. However, information concerning the frequency of this mutation among the Thai population spanning all parts of the country remains limited. A study of 525 individuals from the northern population with low MCV (< 80 fL), Hb A2 < 3.5%, Hb E + A2 < 25%, and no common α^0^-thalassemia (--^SEA^ and --^THAI^) reported that the frequency of --^CR^ deletion was 1.71% in 525^27^. Recently, a frequency study of heterozygous α^0^-thalassemia (--^CR^) in the Thai population from northern, central, northeastern, and southern Thailand with the inclusion criteria of low MCV (< 75fL) reported a --^CR^ deletion frequency of 0.14% (3/2092). Two heterozygous α^0^-thalassemia (--^CR^) individuals were from the northern population and one was from the southern population^14^. In this study, we reported heterozygous α^0^-thalassemia (--^CR^ deletion) with a frequency of 0.2% (2/844), which was found only in two cases from the southern population but none in the other regions. This difference from the previous study was likely caused by the smaller sample size and the difference in inclusion criteria, as a previous study used an MCV cut-off value lower than 75 fL, increasing the probability of heterozygous α^0^-thalassemia selection and excluding some α^+^-thalassemia mutations^28^. However, our study followed the national guidelines for the prevention and control program of thalassemia and used an MCV cut-off value lower than 80 fL, which reflected the frequency of both α^0^-thalassemia and α^+^-thalassemia in the Thai population. Furthermore, a rare α^0^-thalassemia mutation (--^CR^ and --^SA^) has never been investigated in other Southeast Asian countries, such as Myanmar, Laos, Malaysia, Cambodia, and Vietnam. In Southeast Asia, the allele frequency of α^0^-thalassemia (--^SEA^ and --^THAI^) was reported to range from 0.9–11.0% and 0.0–0.1%, respectively. The allele frequency of α^+^-thalassemia was reported as 11.3–20.4% of -α^3.7^, 0.0–2.3% of -α^4.2^, 0.0–3.8% of α^CS^α , and 0.0–1.4% of α^PS^α^29–33^ (Supplementary Table S1).

In conclusion, we are the first to report the frequency of heterozygous α^0^-thalassemia (--^SA^ deletion) as 0.2% (2/844) among the Thai population. The frequency of this mutation was similar to that of two other rare α^0^-thalassemia mutations (--^CR^ and --^THAI^). Thus, to increase the effectiveness of genetic counseling for thalassemia diseases such as Hb Bart’s hydrops fetalis and Hb H disease, we propose that α^0^-thalassemia diagnosis in the routine setting should be identified with at least four mutations, including --^SEA^, --^THAI^, --^CR^, and --^SA^ deletions.

## Supplementary Information

Supplementary Figure 1.

Supplementary Table 2.

## Data Availability

The datasets generated and/or analyzed during the current study are available from the corresponding author upon reasonable request.
