# Supplementary material for: Revisiting and updating molecular epidemiology of α-thalassemia mutations in Thailand using MLPA and new multiplex gap-PCR for nine α-thalassemia deletion
Source: Sci Rep. 2023 Jun 17;13:9850. doi: 10.1038/s41598-023-36840-8 (PMC10276873; doi:10.1038/s41598-023-36840-8)
Supplement: Supplementary file 1 — Supplementary Figure 1. [file 41598_2023_36840_MOESM1_ESM.pdf]

Supplementary Figure 1 Original agarose gel electrophoresis results of 21 α-thalassemia genotypes (AI-III).

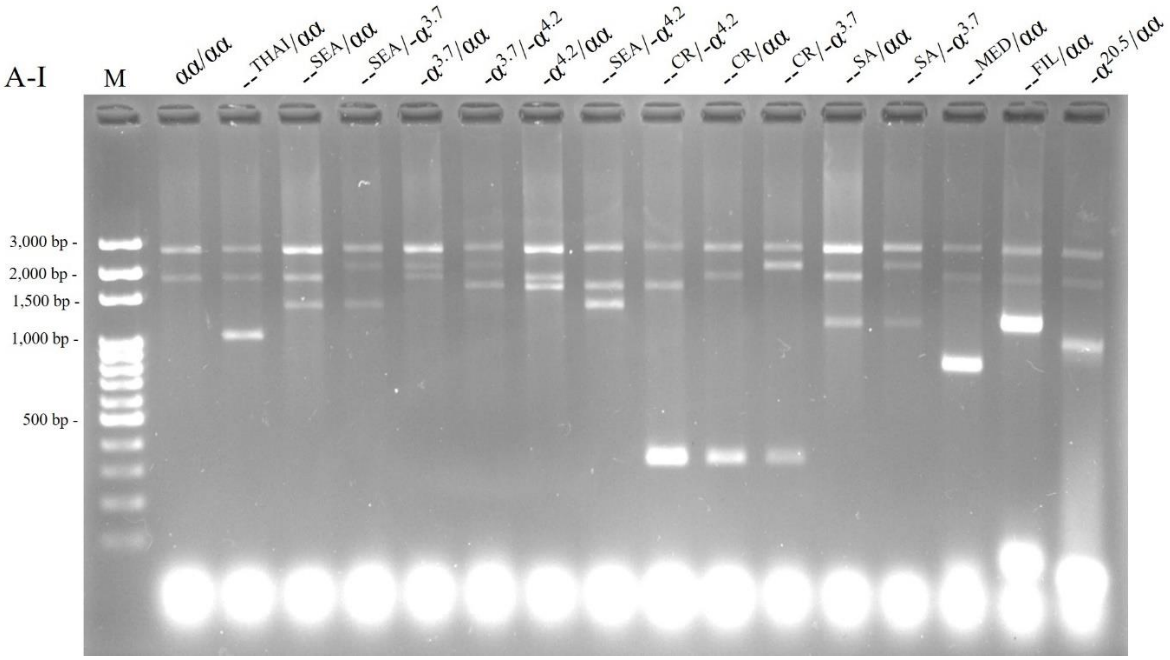

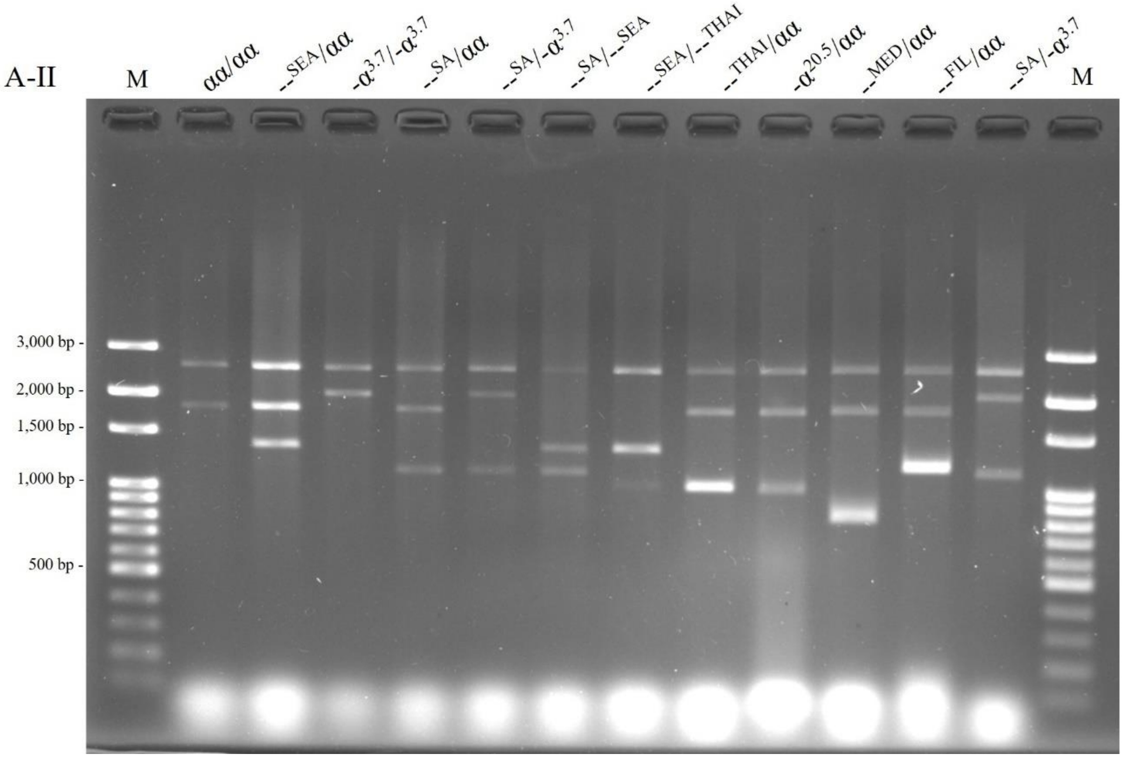

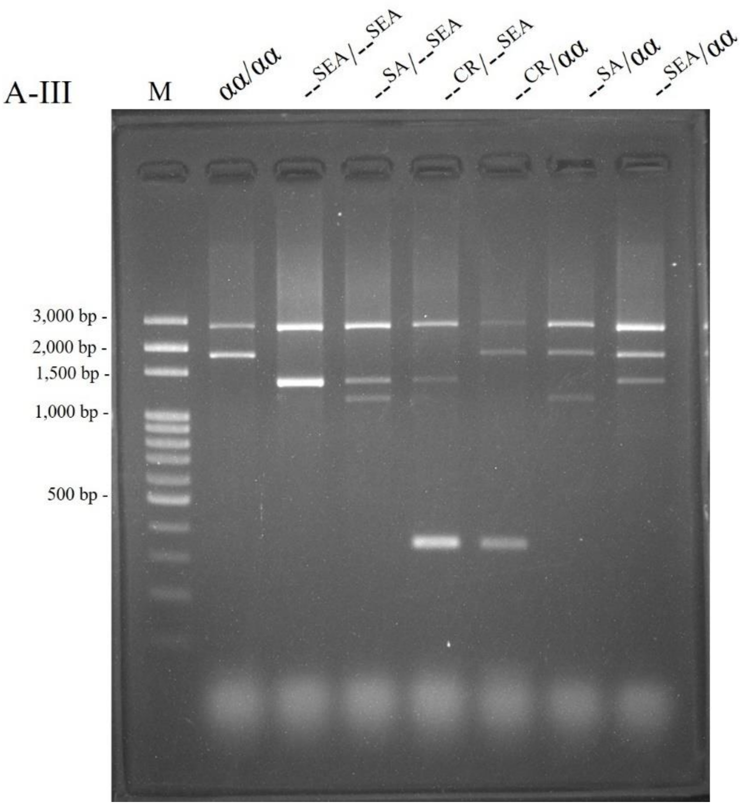
A-III
M
αα/αα
--SEA/--SEA
--SA/--SEA
--CR/--SEA
--CR/αα
--SA/αα
--SEA/αα
3,000 bp -
2,000 bp -
1,500 bp -
1,000 bp -
500 bp -
