# Supplementary material for: Revisiting and updating molecular epidemiology of α-thalassemia mutations in Thailand using MLPA and new multiplex gap-PCR for nine α-thalassemia deletion
Source: Sci Rep. 2023 Jun 17;13:9850. doi: 10.1038/s41598-023-36840-8 (PMC10276873; doi:10.1038/s41598-023-36840-8)
Supplement: Supplementary file 2 — Supplementary Table 2. [file 41598_2023_36840_MOESM2_ESM.pdf]

Table S1 Gene frequency of common α-thalassemia from country nearby Thailand in Southeast Asia. ND not determined

Genes	Myanmar (n=916)	Laos (n=173)	Vietnam (n=1,261)	Cambodia (n=1,631)	Malaysia (n=680)	**Southeast Asia**
	[29]	[30]	[31]	[32]	[33]	[29-33]
$--^{SEA}$	0.0093	0.1098	0.0301	0.0100	0.0662	0.0093-0.1098
$--^{THAI}$	ND	0.0000	0.0000	0.0000	0.0007	0.0000-0.0007
$-\alpha^{3.7}$	0.2036	0.1127	0.1630	0.1670	0.1706	0.1127-0.2036
$-\alpha^{4.2}$	0.0011	0.0231	0.0000	0.0060	0.0110	0.0000-0.0231
$\alpha^{CS}\alpha$	0.0000	0.0347	0.0246	0.0380	0.0147	0.0000-0.0380
$\alpha^{PS}\alpha$	0.0000	0.0058	0.0016	0.0140	ND	0.0000-0.0140
